# Impact of Temporary Opening Using a Stent Retriever on Clinical Outcome in Acute Ischemic Stroke

**DOI:** 10.1371/journal.pone.0124551

**Published:** 2015-04-16

**Authors:** Dongbeom Song, Ji Hoe Heo, Dong Ik Kim, Dong Joon Kim, Byung Moon Kim, Kijeong Lee, Joonsang Yoo, Hye Sun Lee, Hyo Suk Nam, Young Dae Kim

**Affiliations:** 1 Department of Neurology, Yonsei University College of Medicine, Seoul, Korea; 2 Department of Radiology, Yonsei University College of Medicine, Seoul, Korea; 3 Department of Biostatistics, Yonsei University College of Medicine, Seoul, Korea; University Medical Center (UMC) Utrecht, NETHERLANDS

## Abstract

**Background:**

Stent retriever has a distinct ability to restore blood flow temporarily before achieving final reperfusion. There has been a limited report regarding the clinical impact of it. We investigated if temporary opening of occluded vessels using a stent retriever before final reperfusion might improve clinical outcome in acute ischemic stroke patients who received the endovascular reperfusion treatment.

**Methods:**

We enrolled consecutive ischemic stroke patients who had an initial occlusive lesion in the anterior circulation and achieved final reperfusion (Thrombolysis In Cerebral Infarction [TICI] ≥2) by endovascular treatment. Temporary opening was defined as the presence of ante grade flow (TICI≥2) during deployment of a stent retriever. Favorable outcome was defined as a modified Rankin scale score≤2 at 90 day.

**Results:**

A total of 98 patients were included in the study and temporary opening was achieved in 49 (50%). Temporary opening was associated with favorable outcome (odds ratio, 7.825; 95% confidence interval, 1.592–38.461; p = 0.011) in the multivariate analysis. The probability of having a favorable outcome tended to decrease as time from onset to final reperfusion increased in patients without temporary opening. However, this trend was not evident in the patient with temporary opening. The beneficial effect of temporary opening on clinical outcome seemed to be present in patients with good collaterals but not in patients with poor collaterals.

**Conclusions:**

Temporary opening of occluded vessel using a stent retriever may be beneficial for improving clinical outcome in acute ischemic stroke patients.

## Introduction

Endovascular treatment has been used to achieve reperfusion in acute ischemic stroke with large artery occlusion [1]. Despite a higher reperfusion rate of endovascular treatment compared to that of intravenous tissue plasminogen activator (IV t-PA), previous trials using endovascular reperfusion treatment (ERT) failed to show clinical benefit [2–4]. Recently, a stent retriever has emerged as a potential ERT modality that may improve clinical outcome [5–10], and the randomized controlled trials which used stent retriever in 81.5%- 100% of their intervention arms succeeded to demonstrate favorable effect of ERT [11–13].

In addition to the other advantages of a stent retriever, such as easy handling and faster reperfusion, it has a distinct ability to restore blood flow immediately with stent deployment before achieving final reperfusion with stent retrieval [5,14,15]. Theoretically, this temporary blood flow restoration with stent deployment could delay irreversible damage before final reperfusion. We hypothesized that it may improve clinical outcome. Therefore, we compared functional outcome between patients with and those without temporary opening among acute ischemic stroke patients with final reperfusion.

## Materials and Methods

### Ethic statement

This study was approved by the Institutional Review Board of Severance Hospital, Yonsei University Health System with a waiver of consent. Patient information was anonymized and de-identified prior to analysis.

### Patients

This was a retrospective observational study. Among a total of 155 patients who received ERT at our stroke center between January of 2009 and June of 2012, we excluded the patients with posterior circulation infarction (n = 25) or bilateral infarctions (n = 3), and those who were lost to follow up at 3 months (n = 8). We also excluded 21 patients who did not achieve final reperfusion (TICI <2). Finally, we enrolled 98 consecutive ischemic stroke patients who had an initial occlusive lesion in the anterior circulation and who achieve final reperfusion by ERT.

### Reperfusion treatment

The reperfusion treatment protocol for our stroke center has been previously reported [16]. Briefly, patients who presented within 3 hours of symptom onset received IV t-PA, and those who presented at between 3 and 6 hours of symptom onset received ERT. Patients with unsatisfactory clinical response to IV t-PA at the end of the infusion were also considered for additional ERT.

The initial modality of ERT was intra-arterial infusion of urokinase until September 2010. Since then, a stent retriever has been the primary option for ERT unless the occlusion site is too distal or the angioarchitecture is too difficult to approach with a stent retriever. For mechanical clot retrieval with a stent retriever, the Solitaire AB Neurovascular Remodelling Device (Solitaire; ev3 Inc., Irvine, CA) was used. Proximal balloon guide catheter was not used. Solitaire device was deployed within the target clot of the occluded artery and retrieved approximately 7 minutes later. If reperfusion was not achieved, mechanical clot retrieval with Solitaire was repeated. The maximum number of Solitaire passes and the adjunctive rescue therapy in case of initial modality failure was determined by discussion of neurointerventionist and stroke neurologist during each procedure. Adjunctive rescue therapy included clot disruption with a microguidewire or snare, intra-arterial urokinase infusion, and forced-suction thrombectomy with the reperfusion catheter of the Penumbra system (Penumbra; Penumbra Inc., Alameda, CA).

### Clinical variables

For each patient, we investigated vascular risk factors such as hypertension, diabetes, hypercholesterolemia, atrial fibrillation, and previous ischemic stroke. Stroke mechanism, based on the Trial of Org 10172 in Acute Stroke Treatment (TOAST) classification, was determined at a regular stroke conference of the stroke neurologists. Initial stroke severity was assessed using the National Institutes of Health Stroke Scale (NIHSS). Hospital stay was defined as the day from ERT to discharge home or transfer to the Department of Rehabilitation. Favorable outcome was defined as a modified Rankin scale (mRS) score≤2 at 90 days after ERT.

### Imaging analysis

The Alberta Stroke Program Early Computed Tomography (CT) Score (ASPECTS) was investigated using baseline non-contrast CT following recently suggested methodology [17]. ASPECTS was dichotomized at 7 (>7 versus ≤7). The degree of collaterals was graded using the American Society of Interventional and Therapeutic Neuroradiology/Society of Interventional Radiology Collateral Flow Grading System on pre-treatment angiography [18]. Good collaterals were defined as having the degree of collaterals ≥2. Reperfusion was graded using the Thrombolysis In Cerebral Infarction (TICI) scale. Temporary opening was defined as the presence of ante grade flow (TICI ≥2) in the angiography just after deployment. Total number and duration of temporary opening were recorded. Symptomatic intracranial hemorrhage (ICH) was defined as local or remote parenchymal hematoma type 2 within 36 hours after ERT with neurological deterioration (a decrease of ≥4 points in the NIHSS).

### Statistical analysis

We used the IBM SPSS Statistics for Windows, Version 20.0 (IBM Corp., Armonk, NY) for statistical analysis. The Chi square test, Chi squared trend test or Fisher’s exact test for categorical variables, and independent sample t-test or Mann-Whitney U test for continuous variables were performed as appropriate. For investigation of independent factors for favorable clinical outcome, we used a multiple logistic regression analysis adjusting covariates that achieved p <0.05 in the univariate analyses. All statistical tests were 2-tailed, and p <0.05 was considered statistically significant.

## Results

### Overall baseline characteristics

The mean age of the 98 patients included in the study was 69.0 ± 10.4 years and 57 (58.2%) were men. The median NIHSS score was 17 (interquartile range [IQR], 13– 20). The mean time from onset to groin puncture and to final reperfusion was 255.0 ± 88.0 and 353.1 ± 105.1 minutes, respectively. Twelve patients (12.2%) experienced symptomatic ICH after ERT.

### The presence of temporary opening and baseline characteristics

Temporary opening of the occluded vessel using a stent retriever was achieved in 49 patients; 45 were treated initially with a stent retriever and 4 were treated initially with Penumbra but switched to a stent retriever. The remaining 49 patients did not achieve temporary opening before final reperfusion; 40 were initially treated with intra-arterial infusion of urokinase; 4 with carotid stent; 2 with stent retriever; 2 with Penumbra; 1 with simple suction.

In the 49 patients with temporary opening, the median number of temporary opening was 1 (IQR, 0–2) and the mean duration of it was 20.1 ± 13.9 minutes. The mean time from puncture to first temporary opening was 59.1 ± 34.9 minutes and the mean time from first temporary opening to final reperfusion was 37.2 ± 31.2 minutes.

The baseline characteristics between the patients with and those without temporary opening are shown in the Table 1. Internal carotid artery (ICA) occlusion was more often in the patients with temporary opening, while second branch of middle cerebral artery (M2) occlusion was more often in those without temporary opening (Table 1). Other baseline characteristics were comparable between the two groups.

**Table 1:** Baseline characteristics and outcomes of patients with and those without temporary opening.

	Temporary opening (-) (n = 49)	Temporary opening (+) (n = 49)	*p*
Age	70.9 ± 10.8	67.2 ± 9.7	0.080
Male sex	28 (57.1)	29 (59.2)	0.838
Hypertension	36 (73.5)	27 (55.1)	0.058
Diabetes	10 (20.4)	16 (32.7)	0.170
Hyperlipidemia	1 (2.0)	4 (8.2)	0.362
Current smoking	9 (18.4)	10 (20.4)	0.798
Atrial fibrillation	28 (57.1)	26 (53.1)	0.685
Previous ischemic stroke	26 (53.1)	26 (53.1)	1
Prior medication			
Antiplatelet	22 (44.9)	17 (34.7)	0.302
Anticoagulant	5 (10.2)	8 (16.3)	0.372
Arterial occlusion site			0.008
Internal carotid artery	21 (42.9)	28 (57.1)	
Middle cerebral artery, M1	15 (30.6)	19 (38.8)	
Middle cerebral artery, M2	13 (26.5)	2 (4.1)	
Initial NIHSS	16.2 ± 5.4	16.4 ± 5.1	0.878
ASPECTS >7	25 (51.0)	24 (49.0)	0.840
Good collaterals	32 (65.3)	25 (51.0)	0.152
TOAST classification			0.685
Cardioembolism	29 (59.2)	26 (53.1)	
Large artery occlusion	12 (24.5)	10 (20.4)	
Negative evaluation	4 (8.2)	5 (10.2)	
More than two causes	4 (8.2)	7 (14.3)	
Stroke of other determined	0 (0.0)	1 (2.0)	
IV t-PA	27 (55.1%)	30 (61.2)	0.539
Onset to final reperfusion, minute	350.8 ± 95.7	355.3 ± 114.6	0.832
Hospital days	8.9 ± 7.5	9.4 ± 7.0	0.770
Symptomatic intracranial hemorrhage	8 (16.3)	4 (8.2)	0.218
Favorable outcome	20 (40.8)	25 (51.0)	0.331

Data are number (%) or mean (±standard deviation).

ASPECTS, Alberta Stroke Program Early Computed Tomography Score; IV t-PA, Intravenous tissue plasminogen activator; TOAST, Trial of Org 10172 in Acute Stroke Treatment; NIHSS, National Institutes of Health Stroke Scale.

### Favorable clinical outcome and the presence of temporary opening

Among our study population, 45 (45.9%) patients achieved favorable outcome. When we investigated the factors associated with favorable outcome, univariate analysis demonstrated that favorable outcome were associated with good collaterals (p < 0.001), lower initial NIHSS (p < 0.001), the absence of hypertension (p = 0.037), atrial fibrillation (p = 0.001), symptomatic ICH (p = 0.001), and ICA occlusion (p < 0.001). Temporary opening with a stent retriever was not associated with favorable outcome (p = 0.311) in the univariate analysis. However, multivariate analysis adjusted for confounding factors revealed that temporary opening with a stent retriever was a significantly associated with favorable outcome, together with M2 occlusion, good collaterals, and onset to final reperfusion time (Table 2).

**Table 2:** Multivariate analysis of the determinants for favorable outcome at 90 day in patients with acute ischemic stroke.

	Odds Ratio (95% CI)	*P*
Age	1.004 (0.941 1.070)	0.910
Male sex	1.249 (0.317–4.923)	0.751
Hypertension	0.906 (0.228–3.591)	0.888
Atrial fibrillation	0.280 (0.073–1.077)	0.064
Arterial occlusion site		
Internal carotid artery	1	
Middle cerebral artery, M1	2.419 (0.625–9.361)	0.201
Middle cerebral artery, M2	34.404 (3.134–377.718)	0.004
Good collaterals	14.266 (3.024–67.308)	0.001
Onset to final reperfusion, per 1 minute	0.991 (0.984–0.997)	0.005
Initial NIHSS	0.903 (0.783–1.043)	0.164
ASPECTS (>7)	3.570 (0.970–13.141)	0.056
Temporary opening	7.825 (1.592–38.461)	0.011

NIHSS, National Institutes of Health Stroke Scale; ASPECTS, Alberta Stroke Program Early Computed Tomography Score; M1, First branch of middle cerebral artery; M2, Second branch of middle cerebral artery.

**Fig 1:**
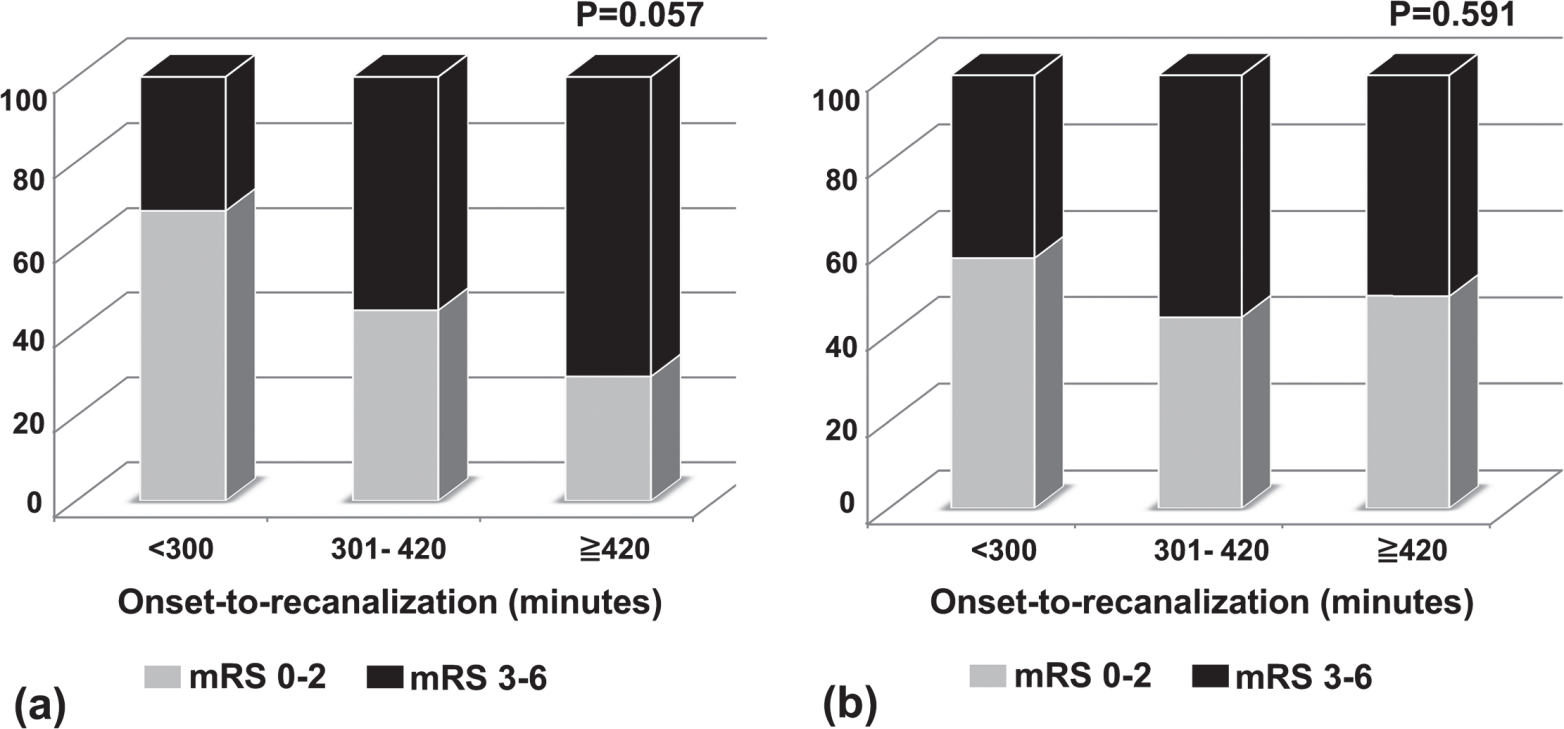
The proportion of favorable outcome at 90 days according to onset to final reperfusion time in patients with temporal opening (a) and without temporal opening (b).

In the analysis between favorable outcome and onset to final reperfusion time, the probability of having favorable outcome tended to be lower as time from onset to final reperfusion is longer in patients without temporary opening (p = 0.057; Fig 1). However, this trend was not evident in the patient with temporary opening (p = 0.591; Fig 1).

**Fig 2:**
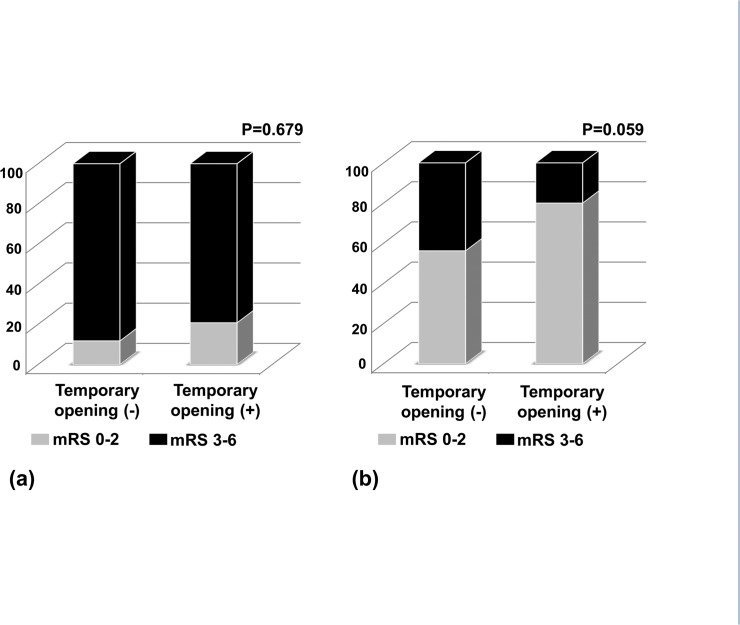
The proportion of favorable outcome at 90 days according to temporary opening in patients with good collaterals (a) and with poor collaterals (b).

Furthermore, we analyzed the relationship between the presence of temporary opening and favorable outcome according to collateral status. Although the beneficial effects of temporary opening on clinical outcome between the two collateral strata were not significantly different in logistic regression model (p = 0.677), it seemed to be present in patients with good collaterals (Odd ratio, 3.111; 95% Confidence interval 0.934 – 10.364; p = 0.059; Fig 2) but not in patients with poor collaterals (Odd ratio, 1.974; 95% Confidence interval 0.335 – 11.634; p = 0.679; Fig 2).

## Discussion

This study showed that temporary opening by a stent retriever was associated with favorable outcome in patients with acute ischemic stroke. Temporary opening also attenuated the time dependent decrease of reperfusion benefit. The beneficial effect of temporary opening seemed to be more evident in patients with good collaterals.

Temporary opening was associated with increased favorable outcome on multivariate analysis, although univariate analysis failed to show a significant relationship between temporary opening and favorable outcome. This may be due to the different baseline characteristics between patients with and those without temporary opening. In particular, ICA occlusion that is associated with worse outcome compared to middle cerebral artery occlusion [19,20], was more common in the temporary opening group. It was because we used intra-arterial infusion of urokinase rather than stent retriever, in case of distal M2 occlusion.

With a longer time to reperfusion, a chance to have favorable outcome decreases. This construct was proven in the previous clinical trials using IV t-PA and ERT [21–24]. In one study using ERT, as onset to final reperfusion time (in minutes) increased, there was a 1.5% decrease in the probability to have a good clinical outcome [23]. However, this time dependent decrease of reperfusion benefit was not evident in patients with temporary opening. It implies that temporary opening helped to maintain some ischemic penumbra to survive. Oxygenated blood to the ischemic penumbra by temporary opening may delay the ischemic necrosis [25]. Intermittent restoration of blood flow may also prevent the microvascular occlusion caused by blood stasis or clot fragmentation after large vessel occlusion.

The beneficial effect of temporary opening seemed to be more apparent in patients with sufficient collaterals. Although the deployment of stent retriever can restore blood flow, it is only transient and the increased amount of blood flow is limited. As more than 10ml/100g/min of blood flow is required at least to prevent membrane failure of ischemic penumbra [26], more blood flow supply by collaterals might be helpful. However, as collateral status was not statistically significant modifier of temporary opening on clinical outcome, this result should be interpreted with caution.

Although temporary opening using stent retriever was associated with favorable outcome, this should not mislead to allow any delay of final reperfusion during ERT. Rapid and complete revascularization is a strong determinant for favorable outcome in acute ischemic stroke patients [4,23,27,28]. Onset to final reperfusion time also remained a significant determinant for favorable outcome even after adjusting the beneficial effect of temporary opening in this study. Therefore, final reperfusion should be accomplished as soon as possible regardless of achievement of temporary opening.

Our study has limitations. First, as all the patients with temporary opening were treated with a stent retriever while most of the patients without temporal opening were not, certain unmeasured characteristics of the stent retriever may affect the outcome. However, as only patients who achieved final reperfusion were enrolled in the study, and other important variables that may affect the outcome were adjusted, we believe that temporary opening improved clinical outcome. Second, as the choice of ERT modality was not randomized, there is a possibility of confounding bias. However, treatment allocation was mainly decided by specific time and the baselines characteristics between the patients with temporary opening and those without were comparable except for the initial occlusion site. Multivariate analysis was also performed to adjust for confounding variables. Third, the time dependent decrease of reperfusion benefit in patients without temporary opening and the beneficial effect of temporary opening in patients with good collaterals had the borderline of statistical significance. It may attribute to relatively small sample size of this study. Finally, this study was retrospective analysis of single center data. Inevitable limitations with the study design should be considered when interpreting the study results.

In conclusion, temporary opening of occluded vessel may improve clinical outcome in ischemic stroke patients. Because temporary opening is a unique ability of stent retriever, and all the patients with temporary opening were treated with a stent retriever, our results provide supportive evidence for its use based on the mechanism of this novel device.
